# Preparation of Magnetic Metal-Organic Frameworks@Molecularly Imprinted Nanoparticles for Specific Extraction and Enrichment of Bisphenol A in Food

**DOI:** 10.3390/foods11101408

**Published:** 2022-05-12

**Authors:** Qi Zhang, Haiyang Wang, Yongju Zhang, Zhixiang Xu, Longhua Xu

**Affiliations:** 1 Key Laboratory of Food Processing Technology and Quality Control in Shandong Province, College of Food Science and Engineering, Shandong Agricultural University, Tai’an 271018, China; 15650452276@163.com (Q.Z.); ocean5416@163.com (H.W.); zhixiangxu@sina.com (Z.X.); 2 Shandong Institute for Product Quality Inspection, Jinan 250102, China; zhangyongju144@163.com

**Keywords:** magnetic solid phase extraction, metal-organic framework, molecularly imprinted polymer, bisphenol A

## Abstract

Metal-organic frameworks (MOFs) with systematically tailored structures have been suggested as promising precursors to the preparation of diverse functional materials. Herein, a facile and versatile layer-by-layer strategy without any special surface modifications has been proposed for the preparation of magnetic metal-organic frameworks (MMOFs) supported molecularly imprinted polymer nanoparticles (MMOFs@MIP), which are based on a magnetically susceptible core conjugated with an imidazole-derived self-assembled layer and a silane-based imprinted shell. The obtained MMOFs@MIPs, which integrated the advantages of Fe_3_O_4_, MOFs, and MIPs, were characterized and exhibited good magnetic properties, a rapid mass transfer rate, and an excellent adsorption selectivity as well as capacity for the targeted molecular - bisphenol A (BPA). Moreover, the MMOFs@MIPs were employed as adsorbents in magnetic solid phase extraction (MSPE) to selectively bind and rapidly separate BPA from real samples with satisfactory recoveries ranging from 88.3% to 92.3%. More importantly, the desirable reusability of MMOFs@MIP was also evaluated, and the recoveries still maintained above 88.0% even after five re-use cycles. Furthermore, combined with high-performance liquid chromatography (HPLC) analysis, a novel MSPE-HPLC method was developed, enabling the highly selective and sensitive detection of BPA in a wide linear range of 0.5–5000 μg L^−1^ with a low limit of detection (LOD) of 0.1 μg L^−1^. This work contributes a promising method for constructing various functional nanoparticles @MOFs@MIP hybrid materials for applications in many different fields.

## 1. Introduction

Bisphenol A is an important industrial monomer that is commonly used as a surfactant and a plasticizer in the manufacturing of polycarbonate plastics and epoxy resins and is applied to the coating of metal surfaces in contact with food and thermal paper [1]. The incomplete polymerization or polymer degradation of BPA allow it to easily migrate and be widely distributed in environmental matrices and food samples. BPA has been proved to be a representative endocrine disruptor that can cause serious damage to the reproductive, nervous, and immune systems and is closely related to many malignant tumors even with a low exposure dose [2,3]. Due to its health risks for humans and other organisms, BPA pollution has received tremendous attention worldwide. While limited to a low concentration, developing low-cost and efficient adsorption materials for monitoring BPA in the environment and food samples is of great significance.

Metal-organic frameworks (MOFs) with systematically tailored structures have been suggested as promising precursors to the preparation of diverse functional materials. Magnetic metal-organic frameworks (MMOFs), which inherit the advantages of magnetic materials and MOFs, have attracted enormous attention in analytical chemistry due to their easy access, super-paramagnetism, rapid adsorption/separation, and reusability. To date, numerous MMOFs have been synthesized using various approaches including embedding, encapsulation, mixing, or layer-by-layer assembly [4], and their intriguing properties facilitated their application as effective adsorbents in sample collection and pre-enrichment, solid-phase extraction, and solid-phase microextraction in recent years. For instance, Yan and co-workers synthesized magnetic MIL-101 microcrystals by physically mixing MIL-101 and silica-coated Fe_3_O_4_ microparticles under ultrasonication and then used the resulting particles for magnetic solid-phase extraction of trace polycyclic aromatic hydrocarbons in water samples [5]. Subsequently, Li’s team reported an embedding method for the fabrication of hybrid MMOF-5s via chemical covalent bonding between amino functionalized Fe_3_O_4_ nanoparticles and the surface of an MOF-5 for magnetic separation and the enrichment of polycyclic aromatic hydrocarbons and gibberellic acid from environmental, food, and plant samples [6]. An encapsulation strategy was adopted for the synthesis of a core–shell structured MMOF nanocomposite (Fe_3_O_4_@ZIF-8) for the elimination of U(VI) and Eu(III) from the environment [7]. The particles were synthesized by nucleation through PSS-modified Fe_3_O_4_ with a negative charge to attract Zn^2+^ cations to from a ZIF-8 layer. Fe_3_O_4_@AMCA-MIL53(Al) for the removal of U(VI) and Th(IV) metal ions from aqueous environments was also obtained through this strategy by using alkaline co-precipitation of FeCl_2_ and FeCl_3_ in the presence of AMCA-MIL53(Al) [8]. Recently, the layer-by-layer assembly approach has received considerable attention and has been widely employed to obtain well-defined core–shell-structured MMOFs. For example, Chen et al. synthesized Fe_3_O_4_@MIL-100 (Fe) for the elective capture of phosphopeptides by adding a MOF shell onto Fe_3_O_4_ nanoparticles [9]. Zhang et al. prepared a magnetic bimetallic metal-organic framework with Zr−O and Ti−O clusters for global phosphopeptide enrichment via the coordination of metal ions with carboxyl groups [10].

Despite the fact that a considerable quantity of MMOFs have been developed and exhibit unparalleled advantages as sorbents for the concentration and separation of trace analytes, their intractable or insufficient selectivity is still a great challenge. In order to solve this problem, numerous attempts have been made to chemical post-modification of the as-prepared MMOFs. For example, Ke et al. designed thiol-functionalized Fe_3_O_4_@MOF for the selective removal of Hg^2+^ and Pb^2+^ from wastewater [11], while Xu et al. employed a beta-cyclodextrin-functionalized MMOF to selectively extract prochloraz and triazole fungicides from vegetable samples [12].

However, the selectivity for a specific target that only depends on functional group modification is not highly developed, especially in a complex sample matrix. Thus, developing a simple and generally applicable methodology to improve the selectivity of MMOFs is of great importance. Fortunately, this possibility was enabled by molecular imprinting technology, which has been proven as an efficient and straightforward approach to producing artificial antibody-like materials with specific molecular-recognition sites [13,14,15,16,17]. The resulting molecularly imprinted polymers have been employed in MOF-based adsorption materials to improve their selectivity [18,19,20].

Inspired by these, we demonstrated a facile and versatile strategy for the synthesis of magnetic metal-organic frameworks with molecularly imprinted nanoparticles via an efficient sol–gel molecular imprinting process in the presence of MMOFs. The fabricated magnetic metal-organic frameworks @molecularly imprinted nanoparticles (MMOFs@MIP and Fe_3_O_4_@ZIF-8@MIP) presented a well-bedded core–shell structure, excellent magnetism, good reusability, fast adsorption, and high selectivity for the target molecule. It was successfully used as an adsorbent to extract and concentrate BPA, which is an endocrine disrupter that can be frequently found in environmental media or packaged foods. Furthermore, combined with HPLC analysis, the Fe_3_O_4_@ZIF-8@MIPs were employed as an absorbent in magnetic solid phase extraction (MSPE) for the highly selective and sensitive detection of BPA in food samples. The developed MSPE-HPLC method has a wide linear range of 0.5–5000 μg L^−1^ and a low LOD of 0.1 μg L^−1^ (*S/N* = 3). At three concentration levels of 0.5, 1.0, and 10 μg L^−1^, the satisfactory recoveries ranging from 88.3% to 92.3% were obtained in uncontaminated lemon juice, canned hawthorn, and mineral water samples, indicating the excellent ability of the prepared Fe_3_O_4_@ZIF-8@MIP to recognize BPA in food samples and its potential for application in BPA detection.

## 2. Materials and Methods

### 2.1. Chemicals

3-aminopropyltriethoxysilane (APTES, 98%) and tetraethoxysilane (TEOS, 99%) were purchased from Shanghai Macklin Biochemical Co., Ltd. (Shanghai, China). Bisphenol A (BPA, 99%), bisphenol B (BPB, 99.5%), bisphenol F (BPF, 99%), bisphenol S (BPS, 99%), bisphenol AF (BPAF, 98%), resorcinol (HQ, 99%), and phenol (P, 99.5%) were obtained from Aladdin Industrial Corporation (Shanghai, China). Unless noted otherwise, all chemicals were used as received.

### 2.2. Preparation of Core–Shell MMOF@MIP (Fe_3_O_4_@ZIF-8@MIP)

**Scheme 1:**
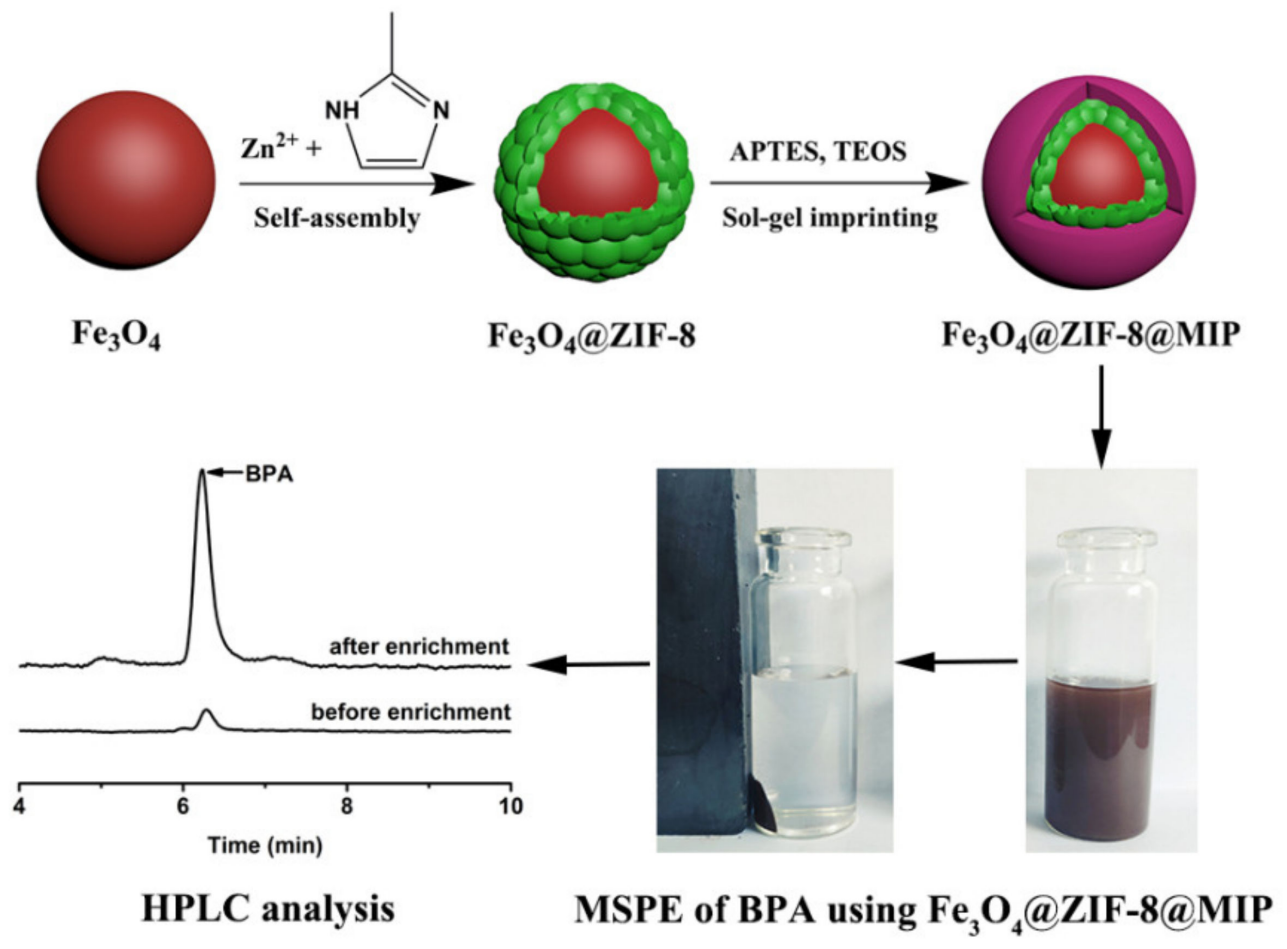
Schematic illustration of the fabrication and application of Fe_3_O_4_@ZIF-8@MIP for extraction and enrichment of bisphenol A.

An Fe_3_O_4_@ZIF-8 was first synthesized using a gentle one-pot self-assembly strategy from a previous report [21,22]. An Fe_3_O_4_@ZIF-8@MIP was fabricated as illustrated in Scheme 1. The pre-assembly solution was prepared by mixing the template BPA (1 mmol) and the functional monomer APTES (4 mmol) in ethanol (30 mL) under gentle stirring for 1 h. Next, Fe_3_O_4_@ZIF-8 (150 mg) as a support was dispersed into the preassembly solution and stirred for 0.5 h. Subsequently, the cross-linker TEOS (12 mmol) and catalyzer HCl (1 mL, 1 mol L^−1^) were successively added dropwise. The pre-polymerization mixture was placed at room temperature for 1 h and then incubated in a water bath at 60 °C for 10 h. The resulting material was washed via Soxhlet extraction in acetic acid and methanol (1:9, *v*/*v*) until no template BPA was detectable by HPLC. Finally, the product was washed with methanol and dried under a vacuum to obtain Fe_3_O_4_@ZIF-8@MIP.

As a control, a non-imprinted Fe_3_O_4_@ZIF-8 (Fe_3_O_4_@ZIF-8@NIP) and a molecularly imprinted polymer coated magnetic Fe_3_O_4_ (Fe_3_O_4_@MIP) were also prepared following the same procedure but in the absence of the template BPA and using Fe_3_O_4_ as a support instead of Fe_3_O_4_@ZIF-8.

### 2.3. Extraction and Detection of BPA in Real Samples

#### 2.3.1. Sample Pretreatment

The lemon juice, canned hawthorn, and mineral water samples were purchased from a local supermarket (Tai’an, China). A reasonable quantity of the aqueous phase in contact with canned foods was filtered through 0.45 μm glass fiber membrane syringe filters; the pH was adjusted to 7 by the dropwise addition of NaOH (1 M) and then stored at 4 °C for further use.

#### 2.3.2. MSPE of BPA from Samples Using MMOF@MIP

The as-synthesized Fe_3_O_4_@ZIF-8@MIP was used as an absorbent for the magnetic solid phase extraction of BPA. Briefly, Fe_3_O_4_@ZIF-8@MIP P (0.5 mg) was added into a volumetric flask and rinsed in sequence with methanol and water. Then, the MMIPMs were separated under an external magnetic field and the supernatant was discarded. Subsequently, 50 mL of the sample extract solution was added into the volumetric flask and mixed via mechanically shaking for 30 min at room temperature, followed by separation with a magnet, and then eluted with a mixture of methanol and acetic acid (3 mL, *v*/*v* = 9:1) under ultrasonic treatment for 30 s. The collected eluent was evaporated to near dryness at a reduced pressure at 50 °C and re-dissolved with methanol to 1 mL for HPLC analysis.

#### 2.3.3. HPLC Analysis

The detection of BPA was conducted via a Shimadzu LC-20AT HPLC system, which consisted of an LC-20AT pump, an RF-10AXL fluorescence detector set to an excitation wavelength of 227 nm and emission wavelength of 313 nm, an SIL-20A automatic sampler with a 20 μL injection loop, an LC workstation for data collection, a CTO-20A column oven set at 35 °C, and a C18 reversed-phase column (250 mm × 4.6 mm, 5 μm, Agilent Technologies, Palo Alto, California, USA) for component separation. The mobile phase was composed of methanol/water (70:30, *v*/*v*) at a flow rate of 1.0 mL min^−1^.

## 3. Results and Discussion

### 3.1. Construction of Core–Shell Fe_3_O_4_@ZIF-8@MIP

**Figure 1:**
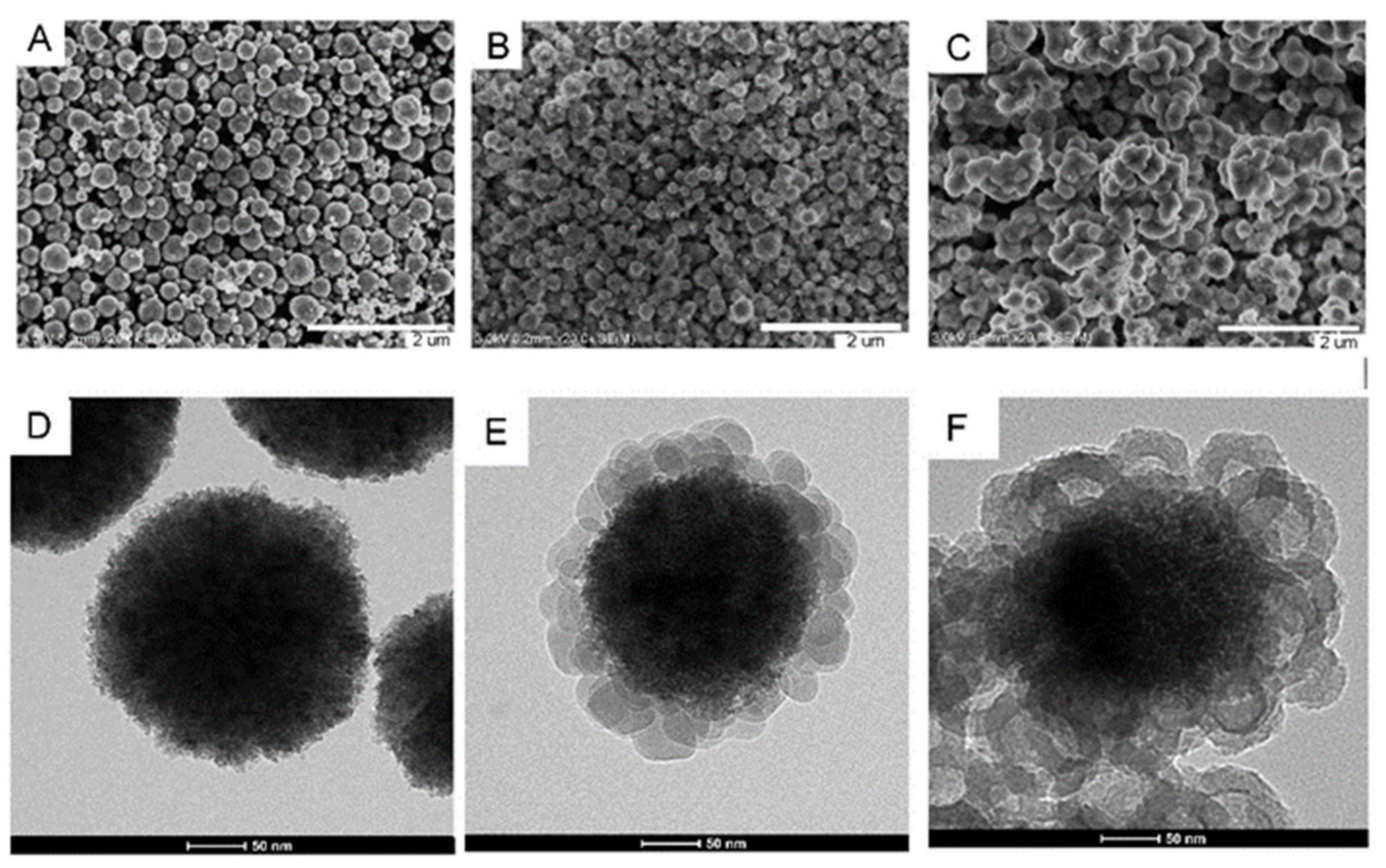
SEM images of Fe_3_O_4_ (**A**), Fe_3_O_4_@ZIF-8 (**B**), and Fe_3_O_4_@ZIF-8@MIP (**C**); TEM images of Fe_3_O_4_ (**D**), Fe_3_O_4_@ZIF-8 (**E**), and Fe_3_O_4_@ZIF-8@MIP (**F**).

The strategy for fabricating MMOFs@MIPs (Fe_3_O_4_@ZIF-8@MIP) is schematically depicted in Figure 1. As a prototypical imidazole-based MOF with outstanding chemical and thermal stability as well as a high porosity and an easy preparation, ZIF-8 was selected as an ideal matrix to develop MMOFs. Carboxylate Fe_3_O_4_ particles were first prepared as a magnetic unit module through a simple solvothermal reaction using trisodium citrate as a stabilizer to obtain a negatively charged Fe_3_O_4_ surface, which favors the attachment of Zn^2+^ cations based on electrostatic interactions to initiate nucleation and growth to produce a ZIF-8 nanocrystal layer. As a result, magnetic Fe_3_O_4_@ZIF-8 was obtained and further employed as a support to fabricate Fe_3_O_4_@ZIF-8@MIP through a molecularly imprinted surface sol–gel process.

### 3.2. Characterization of Fe_3_O_4_@ZIF-8@MIP

#### 3.2.1. SEM and TEM Characterization

The morphologies of the Fe_3_O_4_, Fe_3_O_4_@ZIF-8, and Fe_3_O_4_@ZIF-8@MIP particles were observed by SEM and TEM (Figure 1). As shown in Figure 1A–C, Fe_3_O_4_ (A), Fe_3_O_4_@ZIF-8 (B), and Fe_3_O_4_@ZIF-8@MIP (C) all exhibited nearly spherical shapes with narrow size distributions. In contrast to Fe_3_O_4_, Fe_3_O_4_@ZIF-8 had a rougher surface and a larger particle size. The TEM image (Figure 1D) revealed that the Fe_3_O_4_ nanoparticles were highly monodisperse with an average diameter of 200 nm. Moreover, many closely spaced cubic ZIF-8 crystals were visible on the surface of the Fe_3_O_4_ (Figure 1E), providing a large specific surface area for loading with the MIP layer, and the final product Fe_3_O_4_@ZIF-8@MIP (Figure 1F) had the same core–shell structure as Fe_3_O_4_@ZIF-8. To further confirm the successful synthesis of the Fe_3_O_4_@ZIF-8@MIP, the elemental distribution was also investigated (Figure S1). The image captured from several adhesive Fe_3_O_4_@ZIF-8@MIP particles exhibited a beautiful butterfly shape, indicating that Fe_3_O_4_@ZIF-8@MIP is composed of Fe, N, O, C, Si, and Zn, whereby Fe is mainly concentrated on the inside, in contrast to Si and Zn. The detected Zn, N, and Si signals also demonstrated the presence of ZIF-8 and MIP that was deposited on the entire surface of Fe_3_O_4_@ZIF-8.

#### 3.2.2. FT−IR Measurements

**Figure 2:**
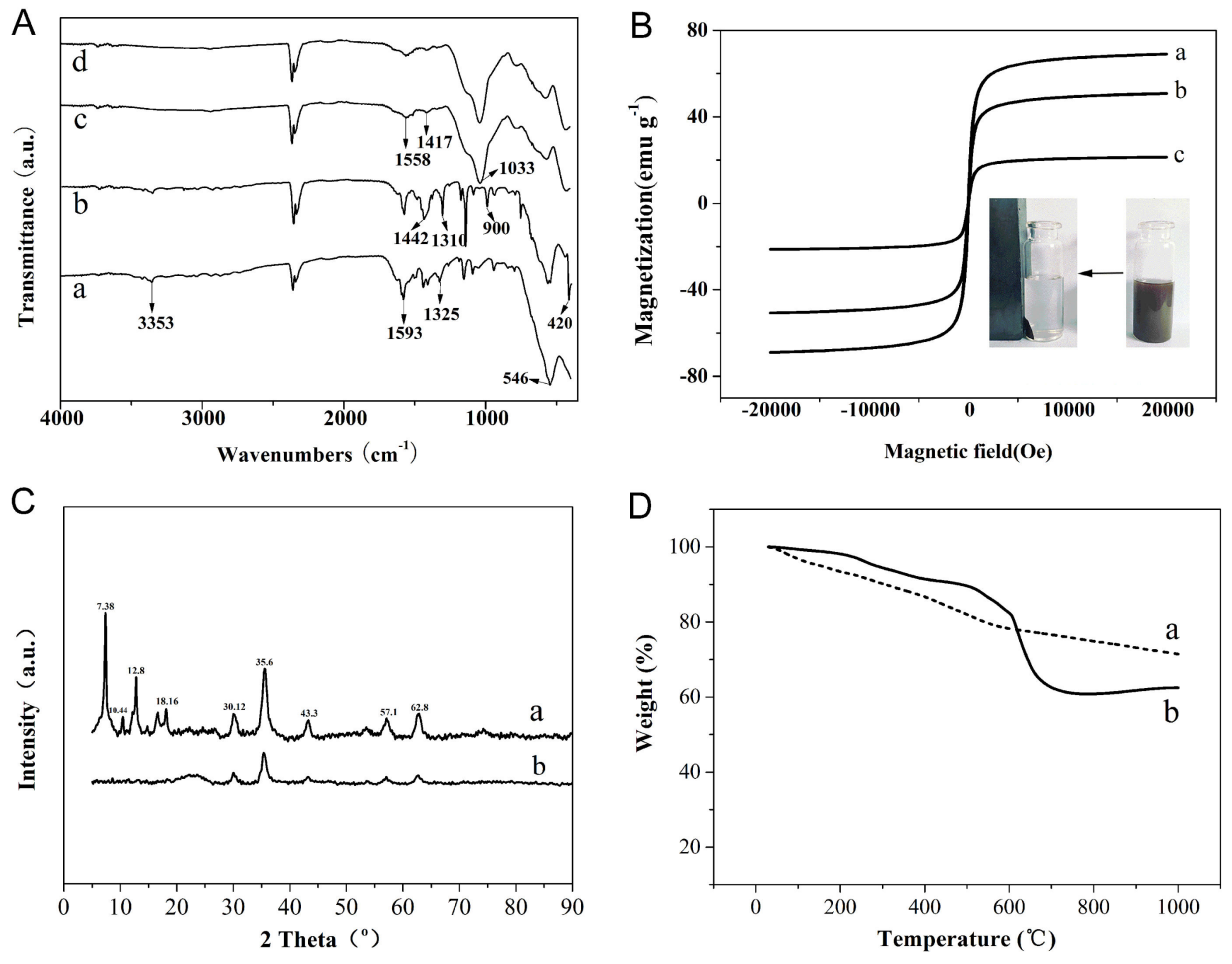
(**A**) FT−IR spectra of Fe_3_O_4_ (a), Fe_3_O_4_@ZIF-8 (b), Fe_3_O_4_@ZIF-8@MIP (c), and Fe_3_O_4_@ZIF-8@NIP (d); (**B**) magnetization curves of Fe_3_O_4_ (a), Fe_3_O_4_@ZIF-8 (b), and Fe_3_O_4_@ZIF-8@MIP (c); insert is a photograph of Fe_3_O_4_@ZIF-8@MIP suspended in solution before and after applying a magnetic field; (**C**) XRD patterns of Fe_3_O_4_@ZIF-8 (a), Fe_3_O_4_@ZIF-8@MIP (b); (**D**) TGA curves of Fe_3_O_4_@ZIF-8@MIP (a) and Fe_3_O_4_@ZIF-8 (b) in air.

As shown in Figure 2A, the functional groups of Fe_3_O_4_ (a), Fe_3_O_4_@ZIF-8 (b), Fe_3_O_4_@ZIF-8@MIP (c), and Fe_3_O_4_@ZIF-8@NIP (d) were analyzed by FT-IR spectra. The strong absorption band at 546 cm^−1^ originated from the Fe−O stretching vibration, and the peaks centered at 3353, 1593, and 1325 cm^−1^ correspond to the characteristic absorption of the −COOH group of trisodium citrate (Figure 2(Aa)), which indicates the successful synthesis of carboxylate Fe_3_O_4_. In Figure 2(Ab), in addition to the characteristic peaks of carboxylate Fe_3_O_4_, the new peaks appearing at around 1442 cm^−1^ and 900–1310 cm^−1^ are attributed to the tensile vibrations and in-plane bending of imidazole rings [23,24], and the absorption peak at 420 cm^−1^ is ascribed to Zn−N stretching vibration, which demonstrates the formation of ZIF-8 on the surface of Fe_3_O_4_. In addition, the characteristic peaks of Fe_3_O_4_@ZIF-8@MIP (Figure 2(Ac)) and Fe_3_O_4_@ZIF-8@NIP (Figure 2(Ad)) were almost the same. For example, the broad and intense absorption peak at 1033 cm^−1^ belongs to the Si−OH stretching vibration, and the peaks nearby at 1558 cm^−1^ and 1417 cm^−1^ are assigned to the stretching and the flexural vibrations of the N-H and −CH_3_ groups [25], indicating the presence of aminopropyl groups. These spectra all confirm the successful encapsulation of Fe_3_O_4_@ZIF-8 by the MIPS via the siloxane copolymerization of APTES and TEOS. At the same time, the similarity of the peaks between Fe_3_O_4_@ZIF-8@MIP (Figure 2(Ac)) and Fe_3_O_4_@ZIF-8@NIP (Figure 2(Ad)) indicates the effective removal of the template-BPA, leaving abundant cavities and accessible sites for BPA adsorption.

#### 3.2.3. VSM Analysis

The field-dependent magnetization curves with no hysteresis loop in Figure 2B show the supermagnetic features of Fe_3_O_4_ (a), Fe_3_O_4_@ZIF-8 (b), and Fe_3_O_4_@ZIF-8@MIP (c) with the determined saturation magnetization values of 69.02, 50.73, and 21.36 emu g^−1^, respectively. The significant decrease in the saturation magnetization values was obviously caused by the layer of ZIF-8 and ZIF-8@MIPs, indicating the successful combination of ZIF-8 or ZIF-8@MIPs and magnetic naparticles. The resulting Fe_3_O_4_@ZIF-8@MIPs still possessed enough magnetic capacity for satisfactory separation. As evident in the insert image, the magnetic Fe_3_O_4_@ZIF-8@MIP can easily form a stable dispersion in ethanol solution without visible sedimentation; can be rapidly separated from the dispersion within a few seconds when a magnetic field is applied; and in turn can be redistributed in solution by simple shaking after the magnetic field is removed, which provides an accessible route for use as an absorbent in target separation or pollutant removal.

#### 3.2.4. XRD Analysis

The crystal structures of synthesized Fe_3_O_4_@ZIF-8 (a) and Fe_3_O_4_@ZIF-8@MIP (b) were confirmed by powder XRD analysis (Figure 2C). The diffraction peaks of Fe_3_O_4_@ZIF-8 displayed at 2θ = 7.38°, 10.44°, 12.8°, and 18.16° are attributed to the (011), (002), (112), and (222) planes, which agrees well with the pure phase of ZIF-8, and the reflection peaks located at 2θ = 30.12°, 35.6°, 43.3°, and 30.12° correspond to the (220), (311), (400), (511), and (440) planes, in tune with the Fe_3_O_4_ lattice, which attests that the growth of ZIF-8 on Fe_3_O_4_ does not impact their respective crystalline integrities_._ Compared to Fe_3_O_4_@ZIF-8 (a), the characteristic diffraction peaks belonging to ZIF-8 were not observed and a new broad peak at 19.04–26.56° appeared in the pattern of the Fe_3_O_4_@ZIF-8@MIPs, which can be ascribed to the coverage effect of MIPs on Fe_3_O_4_@ZIF-8.

#### 3.2.5. TG Analysis

TG analysis was performed to assess the thermal behavior of Fe_3_O_4_@ZIF-8@MIP (a) and Fe_3_O_4_@ZIF-8 (b), as shown in Figure 2D. When heated at temperatures of 25–500 °C, Fe_3_O_4_@ZIF-8 exhibited a gradual weight loss of 18%, resulting from the evaporation of the residual solvent and the pyrolysis of the carboxyl and silicon hydroxyl groups on the surface of ZIF-8 and Fe_3_O_4_, which was much higher than that of Fe_3_O_4_@ZIF-8 (10%) because the MIPs introduced more oxygen-containing groups. When further increasing the temperature to 700 °C, a sharp weight loss (27%) caused by the decomposition of the ZIF-8 framework occurred in Fe_3_O_4_@ZIF-8, whereas only a slight weight loss of 5.4% was observed in Fe_3_O_4_@ZIF-8@MIP, which can be ascribed to the protective effect of MIPs for the ZIF-8 framework. The high decomposition temperature reflects the excellent thermal stability of the Fe_3_O_4_@ZIF-8@ composite.

#### 3.2.6. BET Measurements

The specific surface area and pore characteristics of Fe_3_O_4_@ZIF-8 and Fe_3_O_4_@ZIF-8@MIP have been estimated by N_2_ adsorption and desorption experiments. As shown in Figure S2, a typical IV isotherm with a distinct hysteresis loop was observed for both the Fe_3_O_4_@ZIF-8 and Fe_3_O_4_@ZIF-8@MIPs, indicating their porous nature. Compared with Fe_3_O_4_@ZIF-8 (677.42 m^2^/g), the BET surface area of the Fe_3_O_4_@ZIF-8@ significantly decreased to 4.21 m^2^/g because of the encapsulation of Fe_3_O_4_@ZIF-8 by the MIPs. Using the BJH method, the total pore volume and average pore size was calculated to be 0.047 cm^3^ g^−1^ and 35.98 nm for the Fe_3_O_4_@ZIF-8, as well as 0.036 cm^3^ g^−1^ and 19.48 nm for the Fe_3_O_4_@ZIF-8@MIP.

### 3.3. Adsorption Behaviors of MMOFs@MIP for BPA

#### 3.3.1. Adsorption Kinetic Experiment

**Figure 3:**
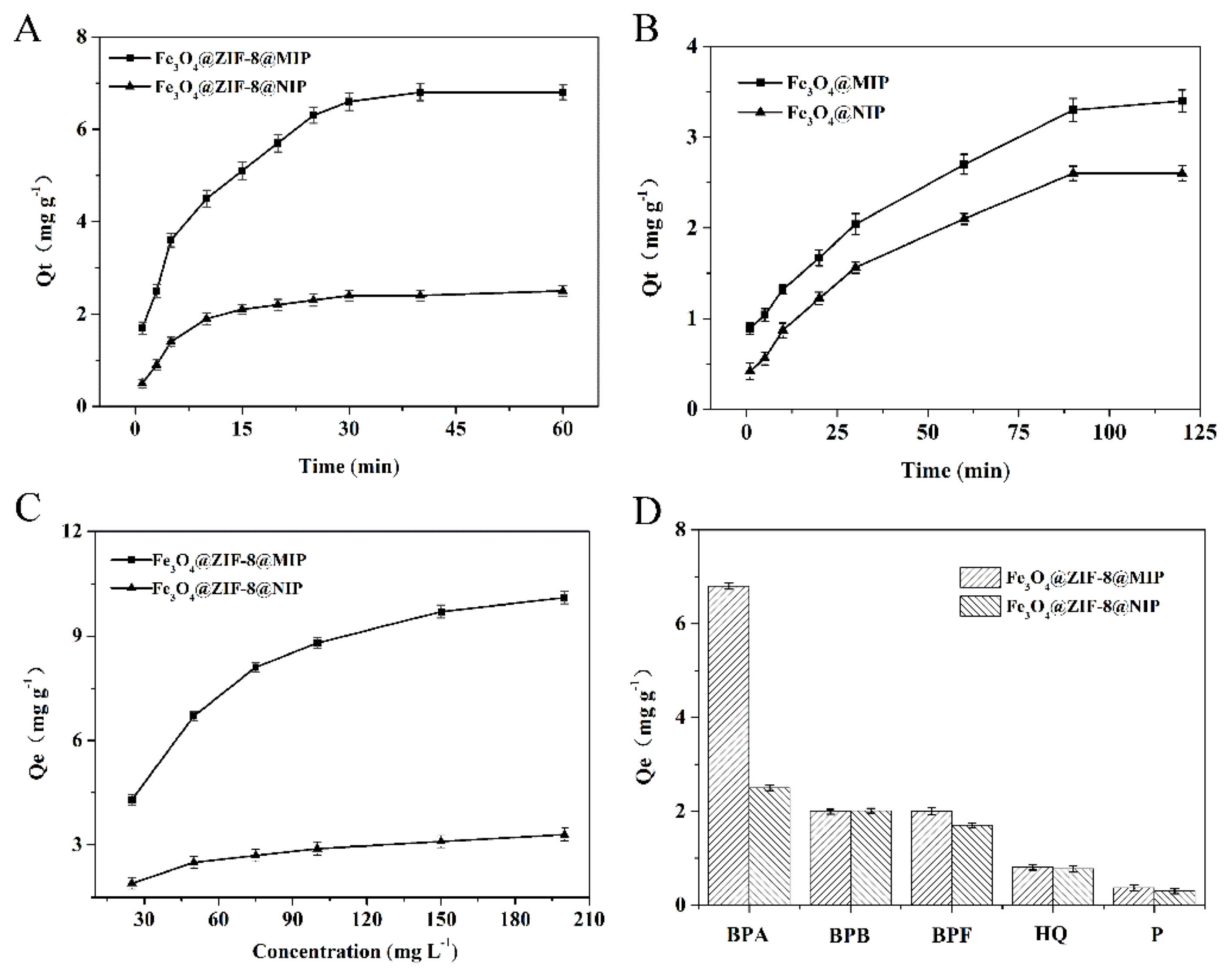
(**A**) Adsorption of BPA (50 mg L^−1^) by Fe_3_O_4_@ZIF-8@MIP and Fe_3_O_4_@ ZIF-8@NIP at different incubation times (1, 3, 5, 10, 20, 25, 30, 40, and 60 min); (**B**) adsorption of BPA (50 mg L^−1^) by Fe_3_O_4_@MIP and Fe_3_O_4_@NIP at different incubation times (1, 5, 10, 20, 30, 60, 90, and 120 min); (**C**) adsorption of BPA by Fe_3_O_4_@ZIF-8@MIP and Fe_3_O_4_@ZIF-8@NIP at different initial BPA concentrations (25, 50, 75, 100, 150, and 200 mg L^−1^); (**D**) absorption of 50 mg L^−1^ initial BPA, as well as its structural analogues BPB, BPF, HQ, and P by Fe_3_O_4_@ZIF-8@MIP and Fe_3_O_4_@ZIF-8@NIP.

The adsorption kinetics of Fe_3_O_4_@ZIF-8@MIPs, Fe_3_O_4_@ZIF-8@NIPs, Fe_3_O_4_@MIPs, and Fe_3_O_4_@NIPs for BPA with an initial concentration of 50 mg L^−1^ were also measured. As shown in Figure 3A,B, benefiting from the presence of imprinted cavities and recognition sites, Fe_3_O_4_@ZIF-8@MIPs not only exhibited a higher adsorption capacity for BPA compared to Fe_3_O_4_@ZIF-8@NIPs (Figure 3A) but also, more importantly, showed a remarkably increased mass transfer rate and faster binding kinetics with a threefold shorter adsorption equilibrium time than Fe_3_O_4_@MIPs (30 vs. 90 min, Figure 3B). This can be ascribed to the pores and specific surface area provided by the MOF.

Additionally, the kinetic data were further analyzed using pseudo-first-order and pseudo-second-order kinetic models. The results shown in Figure S3 illustrate that the dynamic BPA-adsorption behavior of Fe_3_O_4_@ZIF-8@MIPs and Fe_3_O_4_@ZIF-8@NIPs better fit a pseudo-second-order rate equation (R^2^ = 0.9963, 0.9844) rather than a pseudo-first-order equation (R^2^ = 0.9552, 0.9707), indicating that the adsorption process is controlled by the joint action of the solid MOF@polymer/liquid BPA solution interface rather than simple diffusion.

#### 3.3.2. Equilibrium Binding Experiment

The static adsorption behavior of Fe_3_O_4_@ZIF-8@MIP and Fe_3_O_4_@ZIF-8@NIP was studied at room temperature with different initial concentrations of BPA (25–200 mg L^−1^), as shown in Figure 3C. As a control, Fe_3_O_4_@MIP and Fe_3_O_4_@NIP were also employed (Figure S4). The adsorption capacity of Fe_3_O_4_@ZIF-8@MIP and Fe_3_O_4_@MIP was found to display an obvious concentration dependence. When incubated with 200 mg L^−1^ of BPA, the maximum Qe of 10.1 mg g^−1^ was reached for Fe_3_O_4_@ZIF-8@MIP, which is higher than the 3.3 mg g^−1^ for Fe_3_O_4_@ZIF-8@NIP based on nonspecific adsorption (Figure 3D). At the same time, due to the absence of MOFs as carriers, the adsorption capacity of traditional magnetic polymer-Fe_3_O_4_@MIP (Figure S4) was far lower than that of the Fe_3_O_4_@ZIF-8@MIP but slightly higher than that of the Fe_3_O_4_@ZIF-8@NIP, suggesting that there are synergetic effects of MOFs and MIPs that improve the adsorption capacity. The desirable adsorption characteristics make Fe_3_O_4_@ZIF-8@MIP an ideal candidate for the development of highly sensitive and selective adsorption and separation materials.

#### 3.3.3. The Selectivity Evaluation for BPA

The selectivity of BPA adsorption by Fe_3_O_4_@ZIF-8@MIP and Fe_3_O_4_@ZIF-8@NIP was investigated. Phenol-containing compounds including BPB, BPF, HQ, and P with the same initial concentration of 50 mg L^−1^ were selected to test the target binding of Fe_3_O_4_@ZIF-8@MIP toward BPA (see Figure S5 for the corresponding chemical structures). Among them, BPA, BPB, and BPF have similar sizes and arrangements of phenol moieties but increasing hydrophobicity due to substitution at the bridging carbon atom; P, BP, and BPA possess varying numbers and arrangements of phenol groups and aromatic rings. The results are shown in Figure 3D, where the most favorable binding kinetics were observed for imprint molecule-BPA, with a much higher imprinted factor of 2.72 compared to its structural analogues, with values of 1.0 for BPB, 1.18 for BP, 1.05 for HQ, and 1.23 for P. This selectivity can be attributed to the imprinting process that left more specific cavities and recognition sites for the BPA template. In addition, Fe_3_O_4_@ZIF-8@NIP favored the adsorption of BPA, BPB, and BPF rather than the smaller analytes HQ and P, suggesting that silica alone does have some affinity for bisphenol species and that the adsorption process was controlled by the comprehensive effect of the ZIF-8 and MIP layers.

### 3.4. MSPE of BPA Using Fe_3_O_4_@ZIF-8@MIP

**Figure 4:**
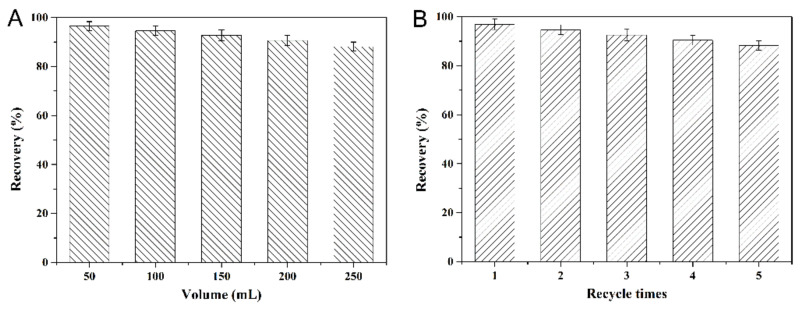
(**A**) Recovery rates of BPA in different sample volumes; (**B**) reusability of the Fe_3_O_4_@ZIF-8@MIP for BPA adsorption.

**Figure 5:**
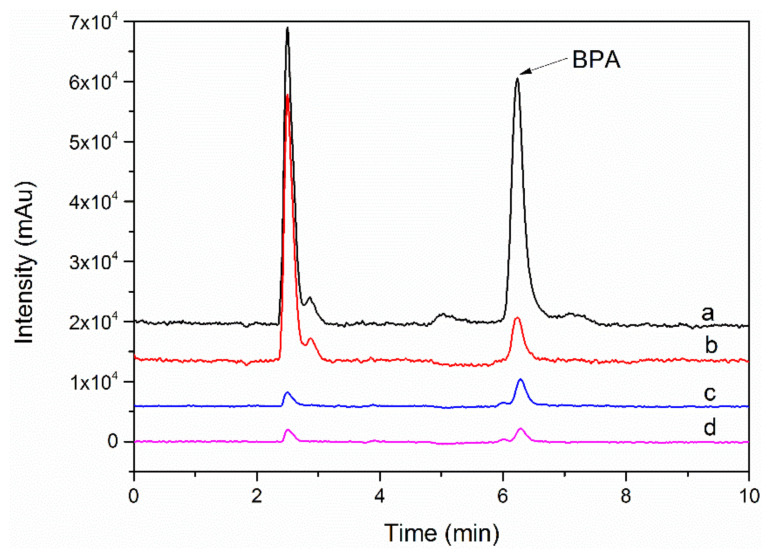
HPLC chromatograms of BPA after enrichment by Fe_3_O_4_@ZIF-8@MIP (a), Fe_3_O_4_@ZIF-8@NIP (b), and its presence in tap water (c) and bottled drinking water (d).

The Fe_3_O_4_@ZIF-8@MIP was employed as a separation tool for the enrichment of trace BPA via a magnetic solid phase extraction, and its application feasibility was studied. As an important parameter, the enrichment efficiency of Fe_3_O_4_@ZIF-8@MIP in different volumes (50, 100, 150, 200, and 250 mL) of solution containing 10 nmol BPA was investigated as shown in Figure 4A. High recovery rates of 88.1–96.5% were obtained, which implies that Fe_3_O_4_@ZIF-8@MIP has good enrichment capabilities for BPA at trace levels. In addition, the enrichment efficiencies of Fe_3_O_4_@ZIF-8@MIP and Fe_3_O_4_@ZIF-8@NIP were also evaluated using 0.5 mg L^−1^ BPA. As shown in Figure 5, the peak intensity after enrichment by Fe_3_O_4_@ZIF-8@MIP (a) was obviously greater than that of Fe_3_O_4_@ZIF-8@NIP (b), indicating the excellent enrichment selectivity of Fe_3_O_4_@ZIF-8@MIP for BPA.

Reusability is another highly desired feature for commercial applications, so the reusability of Fe_3_O_4_@ZIF-8@MIP for 50 μg L^−1^ BPA was evaluated in five consecutive adsorption–desorption cycles, using methanol and acetic acid (3 mL, *v*/*v* = 1:9) as the regeneration agent. As depicted in Figure 4B, after five reuse cycles, the absolute recovery descended to 88% but remained above 90% for the first use, demonstrating its potential recyclability.

### 3.5. MSPE-HPLC for BPA Analysis in Real Samples

Using Fe_3_O_4_@ZIF-8@MIP as an absorbent in MSPE combined with widely accessible HPLC analysis, a highly sensitive and selective MSPE-HPLC detection method for BPA has been proposed with a wide linear range of 0.5–5000 μg L^−1^ and a low LOD of 0.1 μg L^−1^ (*S/N* = 3).

In order to evaluate its reliability and practicability, the developed MSPE-HPLC method based on the Fe_3_O_4_@ZIF-8@MIP was used to extract and determine BPA in real samples including tap water, mineral water, bottled drinking water, lemon juice, and canned hawthorn, among which tap water (Figure 5c) and bottled drinking water (Figure 5d) had detectable BPA levels of 7.5 ± 0.04 μg L^−1^ and 1.2 ± 0.02 μg L^−1^, respectively. Compared to the glass packaging sample (canned hawthorn), BPA in the plastic packaging samples has an increased probability of being detected. It is worth noting that tap water was also contaminated by BPA at a higher concentration than in bottled drinking water, and the extent of this pollution requires more detailed research to be revealed.

To further verify the feasibility of this method, a recovery experiment was carried out by adding a standard solution of BPA at three concentration levels of 0.5, 1.0, and 10 μg L^−1^ into blank lemon juice, canned hawthorn, and mineral water samples. As shown in Table 1, the satisfactory recoveries ranging from 88.3% to 92.3% with an RSD of less than 3.6% were obtained, indicating the acceptable reliability and usability of this method.

As shown in Table S1, compared with the previously developed HPLC methods [26,27,28] for BPA detection, our proposed method based on Fe_3_O_4_@ZIF-8@MIP exhibited higher sensitivity (lower LOD), broader applications including the applicability for simple water matrices as well as a complex fruit juice or canned fruit matrices (which is attributed to the composite effects and high selectivity of MIPs), accessible pores and a good accumulation of MOFs, and the efficient separation of magnetic Fe_3_O_4_.

## 4. Conclusions

We reported a simple and efficient layer-by-layer strategy for the synthesis of core–shell-structured Fe_3_O_4_@ZIF-8@MIPs without any special requirements for surface modification. The synthesized Fe_3_O_4_@ZIF-8@MIP integrates the desirable features of Fe_3_O_4_, MOFs, and MIPs and thus exhibits a strong magnetic responsiveness, an outstanding porosity, and a satisfactory adsorption selectivity for its target. Combined with HPLC analysis, Fe_3_O_4_@ZIF-8@MIPs can be successfully employed for the extraction and determination of BPA in a wide concentration range (0.5–5000 μg L^−1^) with a low LOD (0.1 μg L^−1^). We proposed a feasible strategy for the construction of multifunctional adsorption materials and illustrate the potential for the tailored application of MOFs in more fields.

## Figures and Tables

**Table 1:** Recovery rates of BPA obtained from spiked real samples using the developed MSPE-HPLC method.

Sample	Spiked (μg L^−1^, n = 3)	Recovery (%, n = 3)	RSD (%)
Lemon juice beverage	0.5	88.5	3.0
	1.0	89.3	2.3
	10.0	89.8	2.1
Canned Hawthorn	0.5	89.3	2.9
	1.0	88.3	3.3
	10.0	89.5	1.9
Mineral water	0.5	91.3	2.9
	1.0	92.3	3.6
	10.0	91.9	2.2

## Data Availability

The datasets generated for this study are available upon request to the corresponding author. The data are not publicly available due to privacy.

## Supplementary Materials

The following supporting information can be downloaded at https://www.mdpi.com/article/10.3390/foods11101408/s1, Figure S1: Elemental distribution maps in Fe_3_O_4_@ZIF-8@MIP; Figure S2: N_2_ adsorption –desorption isotherms of Fe_3_O_4_@ZIF-8 (A) andFe_3_O_4_@ZIF-8@MIP (B); Figure S3: Pseudo-first-order (A) and pseudo-second-order (B) absorption kinetic linear fitting curves of Fe_3_O_4_@ZIF-8@MIP and Fe_3_O_4_@ZIF-8@NIP; Figure S4: Adsorption capacity of Fe_3_O_4_@MIP and Fe_3_O_4_@NIP towards BPA in different initial concentrations (25, 50, 100, 150, and 200 mg L^−1^); Figure S5: Chemical structures of bisphenol A and its analogues used in this study; Table S1: Comparison of the proposed MSPE-HPLC method based on Fe_3_O_4_@ZIF-8@MIP with previously reported methods [29,30,31].
