# Supplementary material for: Preparation of Magnetic Metal-Organic Frameworks@Molecularly Imprinted Nanoparticles for Specific Extraction and Enrichment of Bisphenol A in Food
Source: Foods. 2022 May 12;11(10):1408. doi: 10.3390/foods11101408 (PMC9141622; doi:10.3390/foods11101408)
Supplement: Supplementary file 1 [file foods-11-01408-s001.zip › foods-1651260-supplementary-done.pdf]

foods

Preparation of magnetic metal-organic frameworks@molecularly imprinted nanoparticles for specific extraction and enrichment of bisphenol A in food

Qi Zhang[1], Haiyang Wang[1], Yongju Zhang[2], Zhixiang Xu[1], Longhua Xu[1,*]

[1] Key Laboratory of Food Processing Technology and Quality Control in Shandong Province, College of Food Science and Engineering, Shandong Agricultural University, Tai'an, Shandong 271018, P. R. China; 15650452276@163.com(Q. Zhang); 571252194@qq.com(H.Y. Wang.); zhixiangxu@sina.com(Z.X. Xu);

[2] Shandong Institute for Product Quality Inspection, Jinan, Shandong 250102, P. R. China; zhangyongju144@163.com (Y.J. Zhang)

*Corresponding authors: longhuaxu@sdau.edu.cn (Lp.H. Xu)

1. Experimental section

1.1. Chemicals

Ferric chloride hexahydrate ($FeCl_3 \cdot 6H_2O$, 99%), sodium acetate (NaAc, 99%) and trisodium citrate (Na_3Cit, 99%) were purchased from Tianjin Kaitong Chemical Reagent Co., Ltd (China). Zinc nitrate hexahydrate ($Zn(NO_3)_2 \cdot 6H_2O$, 99%), 2-methylimidazole (2-Hmin, 99%). Unless noted otherwise, all chemicals were used as received.

1.2. Synthesis of magnetic Fe_3O_4@ZIF-8 nanoparticles

Carboxylate Fe_3O_4 particles were first prepared via a simple solvothermal process based on the reduction of $FeCl_3$ with ethylene glycol accompanied by carboxylation with Na_3Cit according to a reported method [21]. Fe_3O_4@ZIF-8 was synthesized by a gentle one-pot self-assembly strategy from a previous report [22]. Briefly, 2 mmol of $Zn(NO_3)_2$ was dissolved in ethanol (20 mL, 50%) acidulated with 0.2 mmol HCl, and the synthesized Fe_3O_4 particles (0.35 g) were added to obtain an even dispersion. Afterwards, 40 mL of 50% ethanol solution containing 20 mmol 2-Hmim was rapidly poured into the above solution to initiate the self-assembly reaction with ultrasound for 10 min and stirring for 20 min at room temperature. The produced precipitates were separated using a magnet, washed with ethanol and water several times, and dried in vacuum at 60℃ to obtain the magnetic *Fe_3O_4@ZIF-8*.

1.3. Characterization of synthesized materials

For structural and morphological analysis of the synthesized materials, a Hitachi S4800 scanning electron microscope (SEM, Japan) was employed at an accelerated voltage of 3 kV and a JEOL JEM-2100F transmission electron microscope (TEM, Japan). The elemental distribution was examined using an Oxford X-Max 100TLE energy dispersive spectroscope (EDS, UK) coupled to a high-angle annular dark-field scanning TEM (HAADF-STEM). A NICOLET-iS10 Fourier transform infrared (FT-IR) spectrometer (Thermo, USA) with KBr pellets was used to investigate the structure information, especially for specific functional groups in the samples. The crystallographic characteristics were evaluated by powder X-ray diffraction (XRD) measurements on a D8 Advance X-ray powder diffractometer (Bruker, Germany), with a Cu Kα radiation source (λ= 1.5406 Å) in the 2θ range of 5°~80°, at a scanning speed of 5°/min. The specific surface area, pore volume and pore size were measured by N_2 adsorption at 25°C on an Autosorb-iQA3200-4 instrument (QuantaTech, USA). The specific surface area was calculated using the Brunauer Emmett Teller (BET) method, the total pore volume and the pore size distribution were assessed using the Barrett-Joyner-Halenda (BJH) approach. The thermal gravimetric analysis was performed on a Naichi STA449F5 TG analyzer (China) at atmospheric pressure in the range of 25°C~1000°C with a heating rate of 10°C min^{-1}. Magnetization curves were recorded on a Quantum Design Squid vibrating sample magnenometer in a magnetic field of −20 ~ +20 kOe at room temperature (VSM, USA). The adsorption capacity was assessed by measured the remaining bisphenol in solution using a Metash UV-5500 UV-Vis spectrometer at 278 nm.

1.4. Adsorption properties of synthesized materials

1.4.1. Adsorption kinetic experiment

10 mg of the synthesized materials (Fe_3O_4@ZIF-8@MIP, Fe_3O_4@ZIF-8@NIP) was incubated in 10 mL of BPA standard aqueous solution with initial concentrations of 50 mg L^{-1} (Co, mg L^{-1}) for different time periods (1 min, 2 min, 5 min, 10 min, 15 min, 20 min, 25 min, 30 min, 40 min, 60 min) at room temperature. After magnetic separation, the remaining BPA concentration in the supernatant (Ct, mg L^{-1}) was determined by UV-vis spectrometry, and the adsorption capacity of the synthesized materials for the target molecule was calculated according to the following equation:

$$\mathrm{Qt} = \frac{(Co-Ct)\,V}{m} \tag{1}$$

where V (mL) and m (g) represent the volume of BPA solution and the mass of the adsorbent, respectively.

Next, in order to evaluate the mass transfer and rate-controlling process, pseudo-first-order and pseudo-second-order kinetic models expressed by Equation (2) and Equation (3) were used to analyze the kinetics data of MMOF@MIP:

$$log(Qe - Qt) = log\,Q\,e - \frac{k_1}{2.303}t \tag{2}$$

$$\frac{t}{Qt} = \frac{1}{k_2 Qe^2} + \frac{t}{Qe} \tag{3}$$

where Qe and Qt are the adsorption capacity at equilibrium time and time t (min), respectively. Values of k_1 (min^{-1}) and k_2 (min^{-1}) are the pseudo-first and pseudo-second-order rate constants, respectively.

1.4.2. Equilibrium binding experiment

The static adsorption test was carried out to evaluate the adsorption capacity of the obtained MMOF@MIP particles. In detail, 10 mg of the synthesized materials (Fe_3O_4@ZIF-8@MIP, Fe_3O_4@ZIF-8@NIP, Fe_3O_4@ZIF-8) was incubated in 10 mL solutions with different initial concentrations (25 mg L^{-1}, 50 mg L^{-1}, 75 mg L^{-1}, 100 mg L^{-1}, 150 mg L^{-1}, 200 mg L^{-1}) of BPA or its analogues (Co, mg L^{-1}) until reaching the adsorption equilibrium at room temperature for 30 min. After magnetic separation, the remaining BPA concentration in the supernatant (Ce, mg L^{-1}) was determined by UV-vis spectrometry, and the equilibrium adsorption capacity (Qe, mg g^{-1}) of the synthesized materials for the target molecule was calculated according to the following equation:

$$\mathrm{Qe} = \frac{(Co-Ce)\,V}{m} \tag{4}$$

where V (mL) and m (g) represent the volume of BPA solution and the mass of the adsorbent, respectively.

1.4.3. Selectivity evaluation

To evaluate the specificity adsorption of the synthesized MMOF@MIP/MMOF@NIP toward BPA, phenol-containing compounds including BPB, BPF, HQ and P were selected as structural analogues of BPA. First, 10 mg MMIPMs and MNIPMs were separately incubated in 10 mL of BPA, BPB, BPF, HQ and P solution with the same initial concentration of 50 mg L^{-1}, respectively.

Then, the mixtures were mechanically shaken for 30 min at room temperature. After that, the mixtures were separated with an external magnetic field and the remaining concentration in the supernatant (Ce, mg L^{-1}) was determined by UV-vis spectrometry. The concentrations of five phenol compounds were also detected by UV-Vis ($\lambda_{(BPB,BPF,HQ,\ P)}$ = 278, 278, 290,270 nm).

2. Results

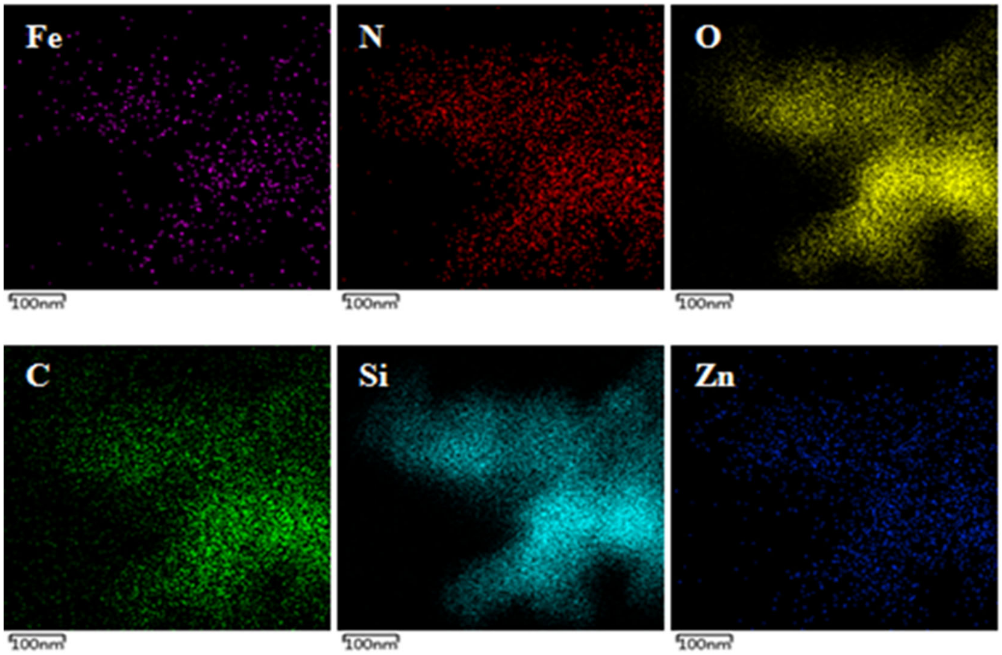

Figure S1. Elemental distribution maps in Fe_3O_4@ZIF-8@MIP.

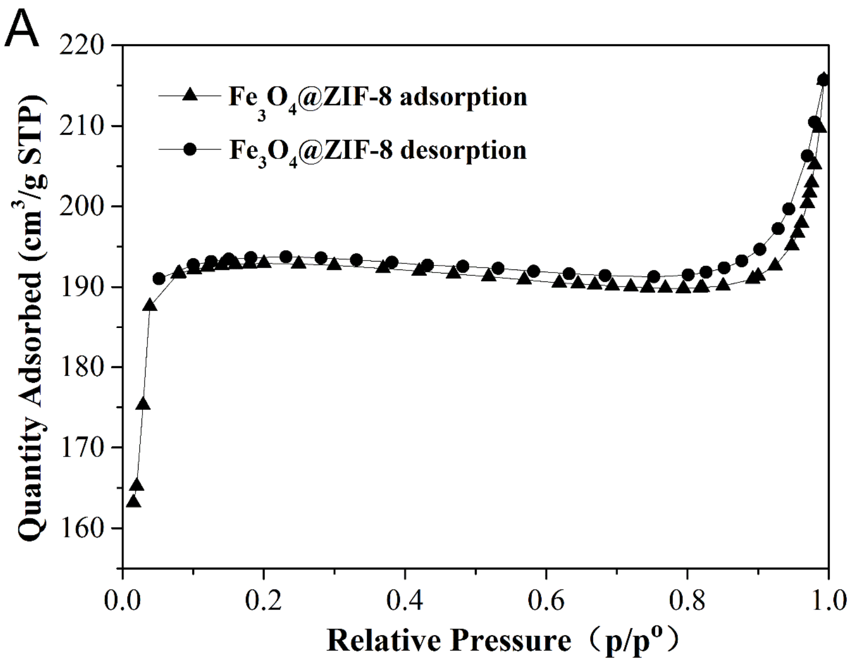

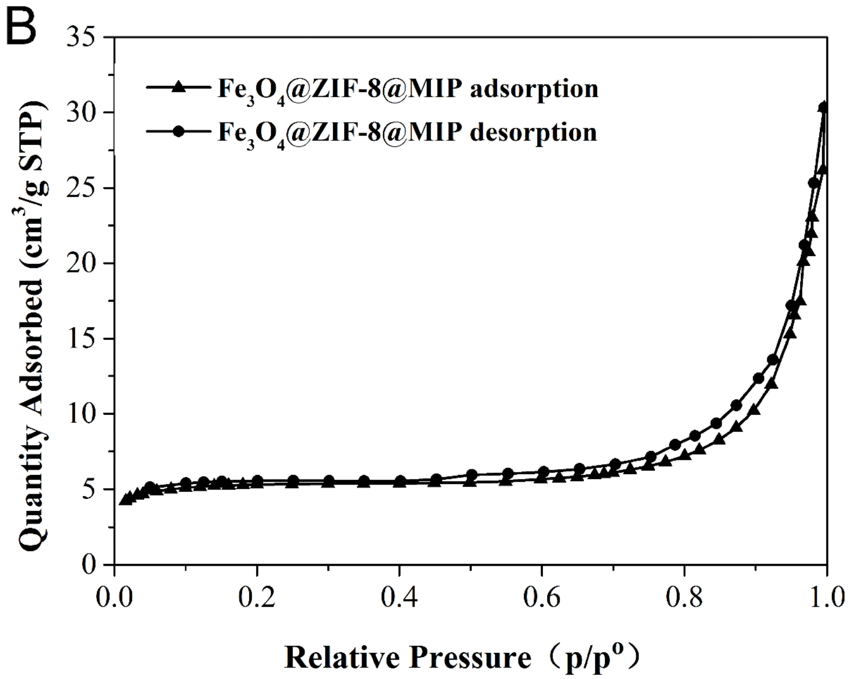

Figure S2. N_2 adsorption-desorption isotherms of Fe_3O_4@ZIF-8 (A) and.

Fe_3O_4@ZIF-8@MIP (B).

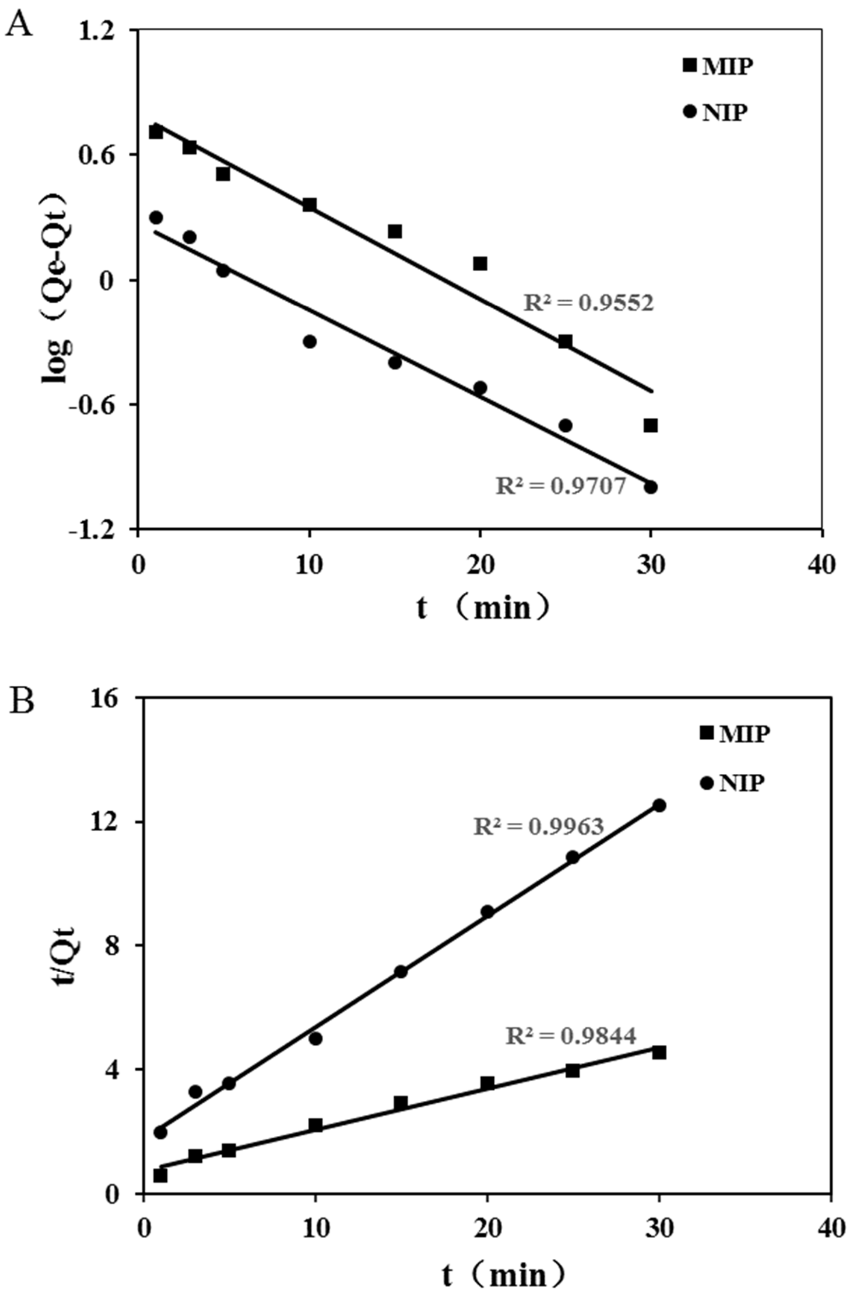

Figure S3. Pseudo-first-order (A) and pseudo-second-order (B) absorption kinetic linear fitting curves of Fe_3O_4@ZIF-8@MIP and Fe_3O_4@ZIF-8@NIP.

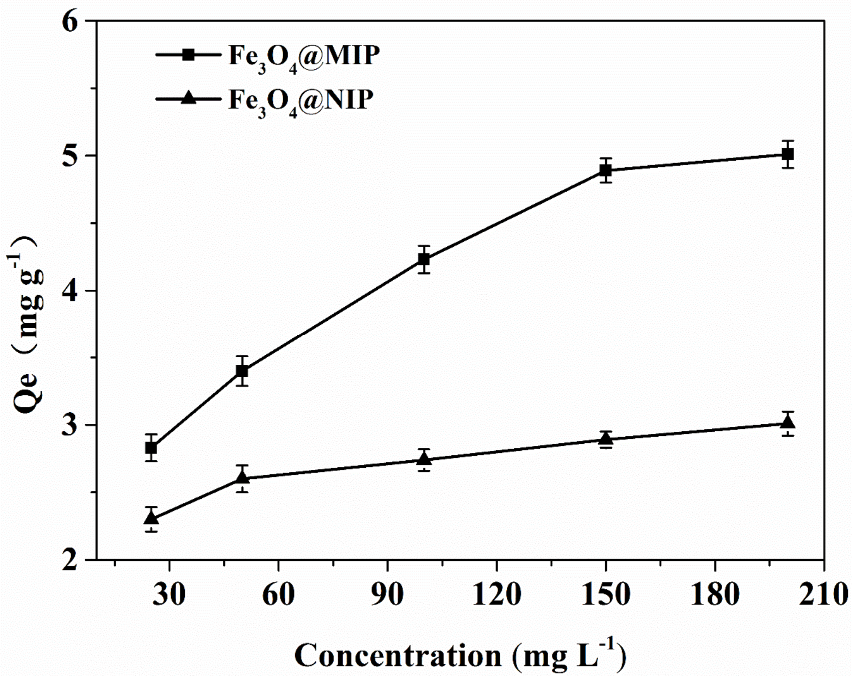

Figure S4. Adsorption capacity of Fe_3O_4@MIP and Fe_3O_4@NIP toward BPA in different initial concentration (25, 50, 100, 150, 200 mg L^{-1}).

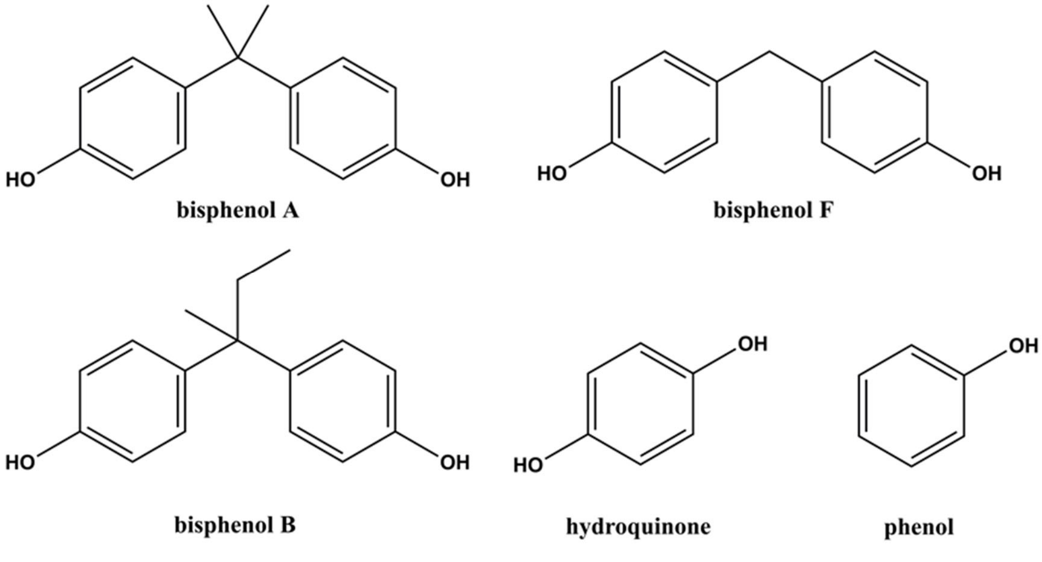

Figure S5. Chemical structures of bisphenol A and its analogues used in this study.

Table S1 Comparison of the proposed MSPE-HPLC method based on Fe_3O_4@ZIF-8@MIP with previously reported methods

Samples	Pretreatment method	Detection method	Recovery (%)	LOD	References
Tap water	MMIP-SPE	HPLC-DAD	90.5~103.7	86.3 ng L^{-1}	Liu et al. [26]
River water	MIP-SPE	HPLC-DAD	76.7~92.3	0.3 ng mL^{-1}	Xu et al. [27]
Tap water	MIP-SPE	HPLC-UV	95.5~106.5	3 ng mL^{-1}	Li et al. [28]
Milk	Fe_3O_4@MIL-SPE	HPLC-DAD	88.17~107.58	0.004~0.108 ng mL^{-1}	Zhou et al. [29]
Water sample and Orange juice	MI-SPE	HPLC-UV	95.0~106.2	0.3 ng mL^{-1}	Wang et al. [30]
Milk	MILs-DLLME	HPLC-UV	98.5%~109.3	5~15 ng mL^{-1}	Zhang et al. [31]
Beverage, Canned Haw-thorn and Mineral water	MMIP-SPE	HPLC-FLD	88.3~92.3	0.1 ng mL^{-1}	This work
